# Practicality of multilayer round window reinforcement in the surgical management of superior semicircular canal dehiscence syndrome: a case report of long-term follow-up

**DOI:** 10.3389/fneur.2024.1393648

**Published:** 2024-06-19

**Authors:** Masafumi Sawada, Han Matsuda, Yasuhiko Tanzawa, Kei Sakamoto, Hiroe Kudo, Masato Nakashima, Tetsuo Ikezono

**Affiliations:** Department of Otolaryngology and Neuro-Otology, Saitama Medical University Hospital, Saitama, Japan

**Keywords:** SCDS, round window reinforcement, long-term effect, superior petrosal sinus, bilateral

## Abstract

Several surgical techniques have been documented for approaching and repairing superior semicircular canal dehiscence syndrome (SCDS). These techniques encompass the trans-middle cranial fossa, transmastoid, endoscopic approaches, and round window reinforcement (RWR). RWR entails the placement of connective tissue with or without cartilage and around the round window niche, restricting the round window’s movement to minimize the 3rd window effect and restore the bony labyrinth closer to its normal state. We employed the multilayer RWR technique, resulting in significant postoperative improvement and long-lasting effects for 3.7 years in 2 cases. Here, we present the clinical findings, surgical procedures, and the effectiveness of multilayer RWR. This technique can be the initial choice for surgical treatments of SCDS due to its high effectiveness, longer-lasting effect, and minimal risk of surgical complications.

## Introduction

Patients with bony labyrinth dehiscence may manifest vestibular and auditory symptoms and cognitive dysfunction, referred to as otic capsule dehiscence syndrome (OCDS) (1). The surgical treatment has demonstrated remarkable efficacy in alleviating these symptoms, rendering OCDS a prominent topic in neuro-otology over the past two decades. Initially identified as superior semicircular canal dehiscence syndrome (SCDS) by Minor et al., SCDS is characterized by a defect in the bone labyrinth of the superior canal (SC), exposing the perilymphatic space to the base of the skull. This SCD acts as the “3rd mobile window” in the inner ear, where external stimuli, such as sound and pressure, can lead to changes in the endolymphatic perfusion of the SC, resulting in distressing symptoms. Patients with SCDS present with a variety of symptoms, with some experiencing more auditory and others experiencing more vestibular complaints. Symptoms may include sound-or pressure-induced vertigo, which is time-locked to the stimulus. Sound-induced vertigo (Tullio phenomenon) involves the induction of disequilibrium and visual field movement (oscillopsia) in response to acoustic stimuli of low frequency. This symptom has a significant impact on patients’ quality of life. Patients may also complain of bone conduction hyperacusis and pulsatile tinnitus.

Surgical intervention for SCDS is generally reserved for patients whose symptoms significantly impact their daily lives. The surgical approaches for addressing SCDS encompass trans-middle cranial fossa, transmastoid, and endoscopic techniques. Various methods have been employed to repair the bone defect, including plugging, resurfacing, and capping (2–4). A relatively recent technique known as round window reinforcement (RWR) has also emerged as a viable option for SCDS treatment. RWR involves the placement of connective tissue with or without cartilage around the round window niche, aiming to bring the bone labyrinth closer to its normal state while limiting the round window’s movement, thus improving symptoms. Despite the limited number of reports on RWR, it has demonstrated utility as a minimally invasive technique for SCDS treatment (5–7).

However, the recurrence of the symptoms post-surgery was the main criticism of RWR; we have developed a multilayer RWR method, which yielded significant symptom improvement that persisted for an impressive 3.7 years, defying the prevailing medical consensus.

The incidence of SCDS has been reported to range from 0.5 to 2% in Europe (8). Its prevalence in Asia remains unknown but is presumed to be rare. In our department, only two cases of SCDS necessitated surgical treatment over the past decade, and both cases underwent multilayer RWR; this report presents the clinical findings, surgical procedures, and postoperative outcomes of these two cases, accompanied by a review of the existing literature.

### Surgical approach

**Figure 1:**
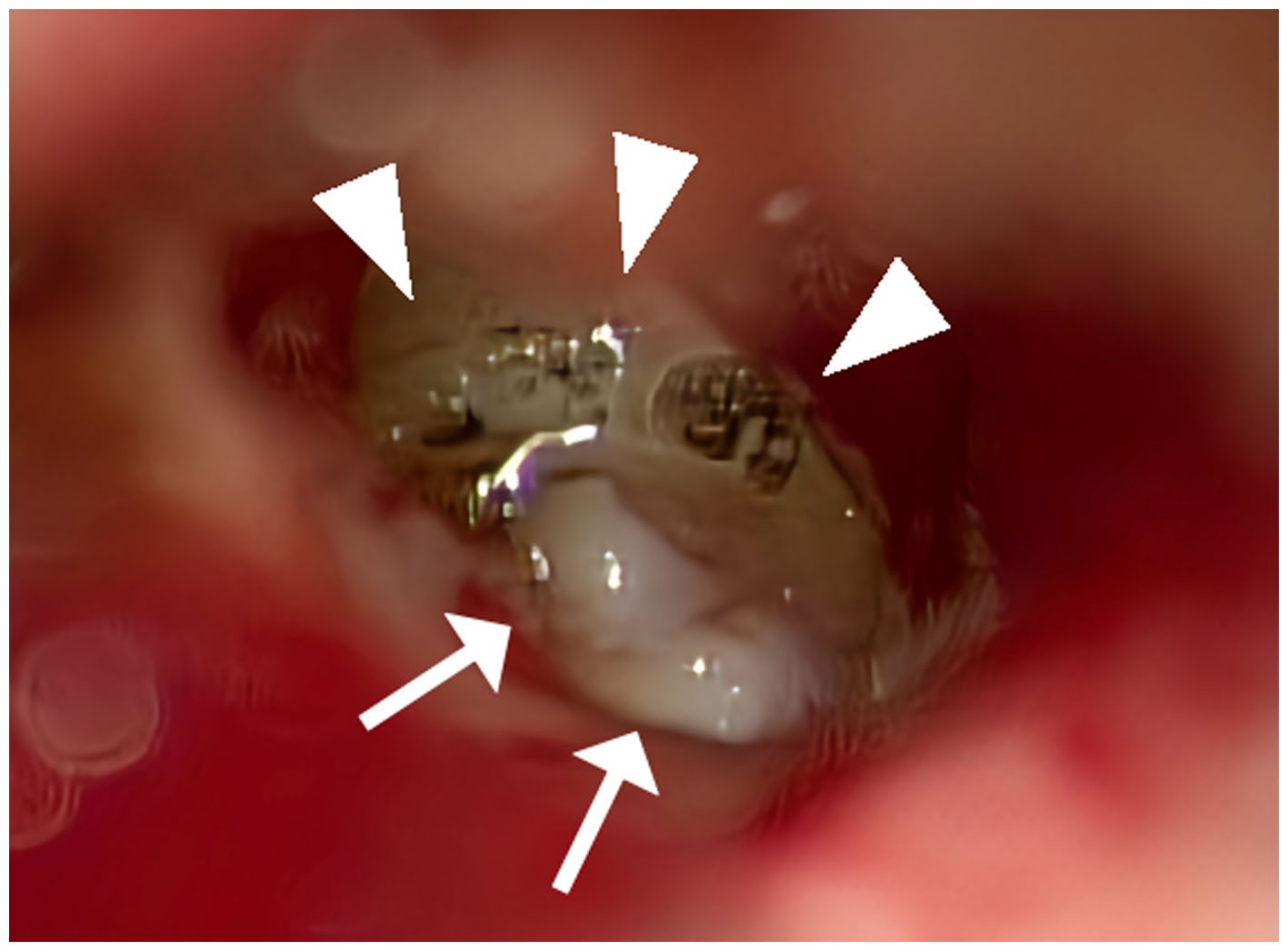
Multilayer RWR techniques. A CO_2_ laser was utilized to remove the mucosa surrounding the round window niche meticulously (allow head). A small piece of thinly sliced cartilage with perichondrium was positioned to fit within the bony overhang of the niche space. Tiny fragments of cartilage were positioned around the initial cartilage to fill the gaps with the bone and stabilize the structure (allow). Thinned connective tissue was applied over the exposed bone surrounding the niche so that it would adhere to the bony surface.

After discussing treatment options, past cure rates, and potential complications, the patients opted for multilayer RWR as the initial surgical procedure, which does not involve a craniotomy or mastoidectomy (Figure 1). Particularly in Case 1, we explained to the patient that SPS-type SCDS complicates securing the visual field during a middle cranial fossa approach. The multilayer RWR technique incorporated modifications of the technique previously reported by Wackym et al. (6). An intra-meatal incision was made, and loose areolar tissue was harvested at the site of the incision. Subsequently, a portion was thinly spread using a compression device and cut into 3–5 mm pieces. Cartilage was harvested with perichondrium from the tragus meticulously thinned with a scalpel, and cut into approximately 2–3 mm diameters in round shapes. Upon accessing the middle ear, a CO_2_ laser (Lumenis Spectra II, Lumenis Inc., San Jose, CA) was utilized to remove the mucosa surrounding the round window niche meticulously. A small piece of thinly sliced cartilage with perichondrium was positioned to fit within the bony overhang of the niche space, facing the perichondrium side toward the RW so that the cartilage would not damage the RW membrane. Tiny fragments of cartilage, approximately 0.25 mm in size, were positioned around the initial cartilage to fill the gaps with the bone and stabilize the structure. Additionally, thinned connective tissue was applied to envelop the first cartilage and these additional pieces, extending over the exposed bone surrounding the niche so that it would adhere to the bony surface. Subsequent layers of additional sliced cartilage were placed on top to prevent migration. Fibrin glue (Beriplast P, CSL Behring, King of Prussia, PA) was used to secure these materials in place (6).

### Clinical assessment

We have evaluated the patients using pure tone audiometry (125 Hz, 250 Hz, 500 Hz, 1 k Hz, 2 k Hz, 4 k Hz, 8 k Hz were measured), ocular vestibular evoked myogenic potentials (oVEMP) in response to air-conducted sound and high-resolution CT scans (HRCT). The patients underwent helical high-resolution computed tomography of the temporal bone with 0.6 mm of the slice thickness in the axial plane. The images were reconstructed in planes parallel to the superior semicircular canal (Pöschl plane) and orthogonal to the canal (Stenver plane), and a head and neck radiologist diagnosed the presence of dehiscence.

We have used three questionnaires: the dizziness handicap inventory (DHI) and the Vertigo Symptom Scale Short Form (VSS-sf), which are widely used. The DHI evaluates handicaps due to dizziness in daily life. An improvement of 18 points or more is considered to represent a minimal clinically important difference (MCID) (9). The VSS-sf was developed to measure symptom frequency over 1 month, with the primary goal of assessing therapeutic effect, and has been used in clinical trials (10, 11). In VSS-sf, the higher the total score from 0 to 60, the more severe the symptoms; a score of 12 or more is considered severe (12). The Niigata PPPD Questionnaire (NPQ), recently developed for PPPD diagnosis, has proven beneficial for this purpose (13). The NPQ asks patients about factors that induce vertigo/dizziness in three categories: upright posture/walking, movement, and visual stimulation. Not only for PPPD diagnosis but in our daily clinical practice, we utilize the NPQ to identify the triggers of a patient’s vestibular symptoms (discussed below). To rule out perilymphatic fistula (PLF), the intraoperative inspection was performed to identify the possible fistula and perilymph-specific protein CTP detection test (14) was performed.

## Cases

### Illustrative case 1

#### Chief complaint: unsteadiness

##### History of present illness

The onset of symptoms began 5 years before the initial visit, with intermittent episodes of unsteadiness (Table 1). These episodes had minimal impact on the patient’s daily life. However, approximately 5 months before the first visit, the symptoms escalated to a point where the patient could not take more than a few steps without experiencing severe unsteadiness. Activities such as using elevators or traveling by car exacerbated these symptoms. Over time, the symptoms progressively deteriorated. Even in the absence of accelerative stimuli from vehicles or elevators, the patient sustained symptoms akin to motion sickness for several months. The episodes of unsteadiness fluctuated, with some days being better than others. Additionally, the patient reported experiencing heightened sensitivity to children’s loud voices, especially in the left ear. The patient’s concentration had significantly declined, making tasks like reading challenging. Due to these symptoms, the patient, a carpenter working at heights, was unable to continue working and had to take medical leave. A HRCT scan raised suspicion of SCD, leading to a referral to our department.

**Table 1:** Patients demographics, history, third window syndrome symptoms, physical findings, and results of diagnostic studies.

Patient (age at surgery)	Sex	Diagnosis	HRCT	Low-frequency air-bone gap	Enhanced VEMP responses	Sound-induced symptom	Preceding trauma	Hearing internal sound	Length of postoperative follow up
Case 1 (43 years old)	M	Lt SPS type SCDS	Lt SPS type	Lt	Lt	Unsteadiness	No	Voice resonant	3.7 years
Case 2 (61 years old)	F	Rt SCDS	Bilateral side	Bilateral	Rt	Vertigo and oscillopsia	Car accident (rear-end collision)	Auto-phony	3.7 years

N/A, not available.

##### Clinical assessment

**Figure 2:**
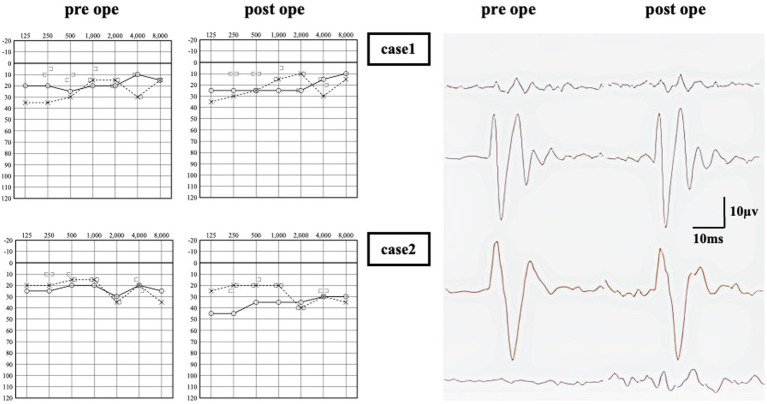
Audiometry of pre and post operation. In Case 1, no significant change in air-conduction (AC) thresholds between pre and post-surgery. An response was observed in the left side oVEMP, and no significant change was observed after surgery. In Case 2, there was a decrease in AC thresholds on the surgical side, resulting in a 15–25 dB air-bone gap in the low frequency range. An enhanced response was observed in oVEMP on the right side only, and no significant change was observed after surgery.

Upon examination, the patient scored 36 points on DHI, 14 points on VSS-sf, and 52 points on NPQ. Neuro-otological examination revealed a left-sided low-frequency air-bone gap of approximately 30 dB on pure tone audiometry (Figure 2). An enhanced response was observed in the left side oVEMP with an asymmetry ratio of 82.5%. HRCT revealed a dehiscence of SPS-type only on the left side in the reconstructions on the Pöschl plane, and surgical intervention was indicated.

##### Surgical approach and outcome

**Figure 3:**
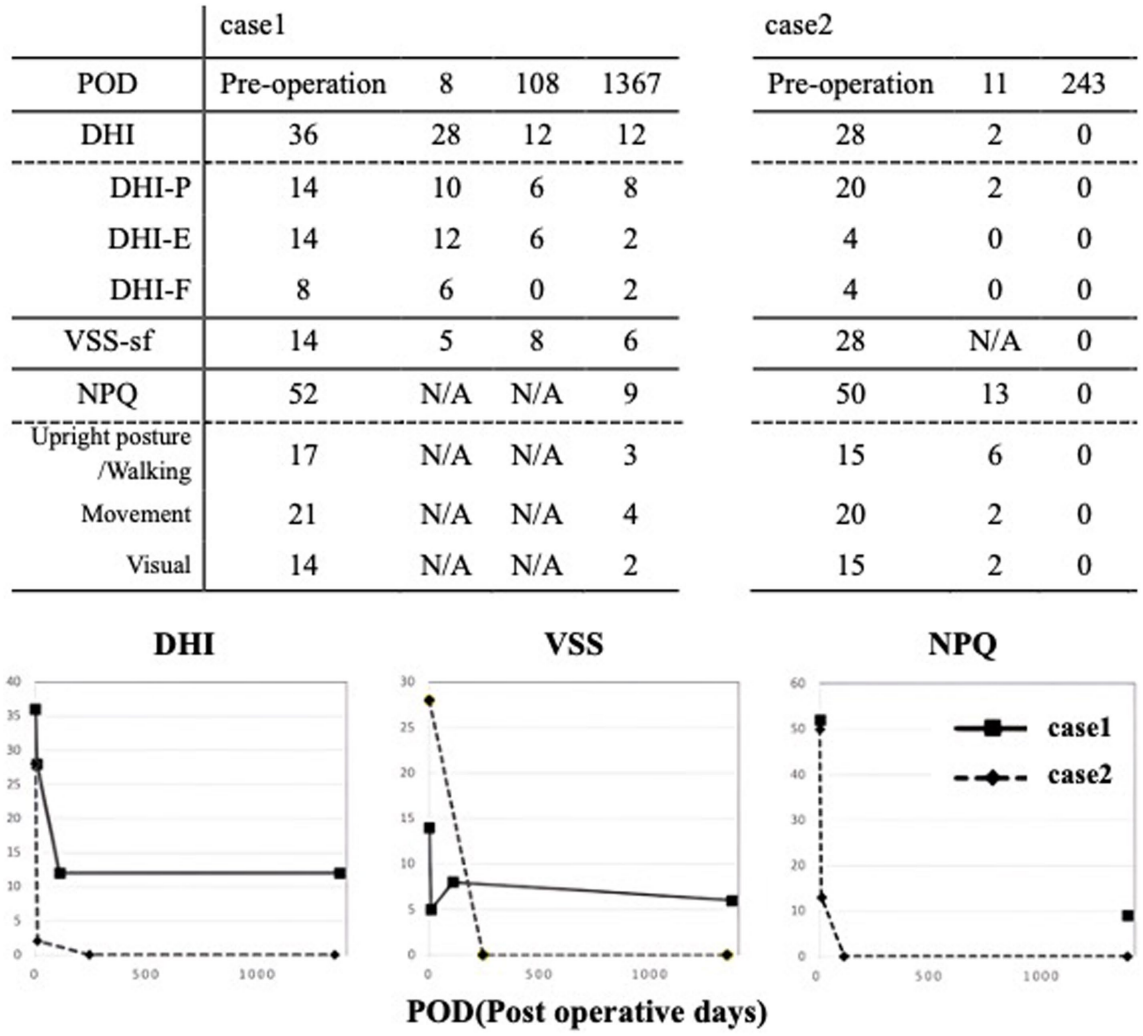
CT imaging. All reconstructed on the Pöschl plane. Case 1: This illustrates slices of the left side, the Pöschl and coronal planes. Notably, only the left side exhibited a dehiscence of SPS type. Case 2: Pöschl plane demonstrating the presence of dehiscence on both sides. Only the right side was symptomatic.

The multilayer RWR technique was chosen as the treatment. No intraoperative fistula was identified, and intraoperative CTP testing was negative. Following the surgery, there was a notable improvement in symptom scores within 8 days. No deterioration was observed up to 1,367 days (3.7 years) postoperatively (Figure 3). Notably, the low-tone air-bone gap and VEMP asymmetry ratio did change following surgery (Figure 2).

### Illustrative case 2

#### Chief complaint: sound-induced vertigo

##### History of present illness

Seven years before the initial consultation, the patient experienced the onset of sound-induced vertigo following a car accident (rear-end collision) (Table 1). Her symptoms notably worsened, primarily when she served as an emcee at sports events held in large venues. This exacerbation occurred in a time-locked manner due to exposure to intense auditory stimuli such as drum rolls, applause, and cheering. During these events, she reported a resonant sensation within her right ear and a sound-induced swaying sensation. Furthermore, she described experiencing severe oscillopsia, wherein the audience appeared to undulate like waves. Additionally, there were episodes during which she perceived spatial disorientation as if the ground and sky had inverted. Her symptoms intensified during airplane take-offs and landings, as well as while ascending in an elevator, leading to severe unsteadiness, necessitating the use of handrails for support.

##### Clinical assessment

**Figure 4:**
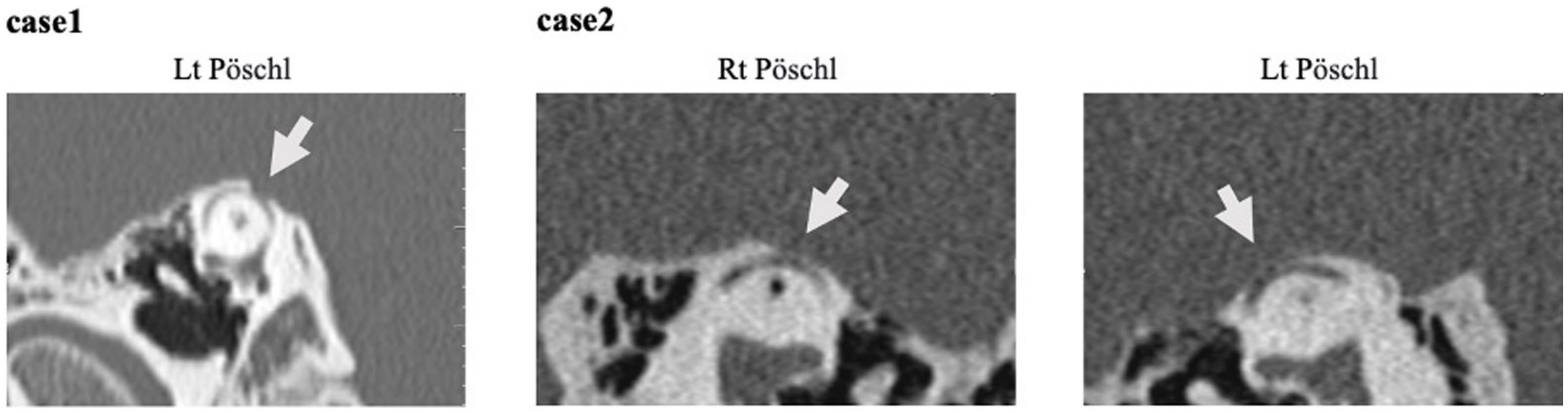
DHI & VSS score of pre and post operation. Following the RWR surgery, there was an improvement in symptom scores. In case 1, DHI from 36 to 12, with no deterioration observed up to 1,367 days (3.7 years). In case 2, DHI from 28 to 2 in 11 days, with no worsening at 1,346 days (3.7 years). VSS remained similar.

At the time of examination, the patient’s symptom scores were as follows: 28 points on DHI, 28 points on VSS-sf, and 50 points on NPQ. Neuro-otological examination revealed a 15 dB air-bone gap (Figure 2). An enhanced response was observed in the right side oVEMP with an asymmetry ratio of 91%. HRCT showed dehiscence of the superior semicircular canal on both sides in the reconstructions on the Pöschl plane (Figure 4). Based on her symptoms and oVEMP results, a diagnosis of right-sided SCDS was made, and surgical treatment was recommended.

##### Surgical approach and outcome

The patient underwent multilayer RWR surgery. No intraoperative fistula was identified, and intraoperative CTP testing was negative. Following the procedure, there was a remarkable improvement in symptom scores within 11 days postoperatively, and no worsening was observed up to 1,346 days (3.7 years) postoperatively (Figure 3). The enhanced oVEMP responses did not show improvement after surgery. The air conduction in pure tone audiometry at lower frequencies increased by 10 dB (Figure 2).

## Discussion

In these 2 cases, characteristic symptoms and findings such as pressure and/or sound-induced vertigo and nystagmus, and air-bone gaps at low tones were identified, and an HRCT scan and VEMP were conducted and confirmed the diagnosis of SCDS. Their symptoms were severe and had a considerable impact on their personal and professional lives.

Patients with larger dehiscence (≥2.5 mm) were significantly more likely to exhibit vestibulocochlear symptoms/signs, enhanced VEMP responses, and objective vestibular findings compared to subjects with smaller bony defects (15). In Case 2, bilateral dehiscences were present, but the symptomatic cause was identified on the right side, with a size of 4.0 mm. The left side, measuring 2.2 mm, was not implicated in the symptoms. These findings are consistent with previous reports.

The RWR technique was developed to alleviate symptoms associated with a third window by modifying the round window (RW) compliance without directly addressing the more challenging-to-access SC. Consequently, the inner ear becomes less susceptible to sudden changes in sound and pressure. The RWR surgery reduced several symptoms of SCDS, including autophony, bone-conduction sensitivity, pulsatile tinnitus, sound or pressure-induced vestibular symptoms, and aural fullness (7, 16).

In the present study, two subjects who underwent multilayer RWR showed marked improvement in their DHI, VSS-sf and NPQ scores shortly after surgery. DHI scores showed significant decreases from 36 to 12 in Case 1 and from 28 to 0 in Case 2, indicating an improvement of more than 18 points (MCID) in both cases. VSS-fs scores, which were above 12 in both cases, indicating severe conditions, demonstrated marked improvements post-surgery, dropping to 6 and 0 points, respectively. The differential diagnosis for SCDS includes varieties of chronic vestibular disease (17), including Meniere’s disease, perilymph fistula, age-related unsteadiness, and persistent postural-perceptual dizziness (PPPD). The NPQ is initially developed to assist in diagnosing PPPD and assesses symptom exacerbation in response to three characteristic factors: upright posture/walking, movement, and visual stimulation. When the cutoff is set at 27 points (total score = 72), the sensitivity of PPPD diagnosis is 70%, and the specificity is 68%. In both cases, NPQ scores exceeded the cutoff of 27 points, 52 points, and 50 points and dropped to 9 and 0, respectively. This does not mean that these cases suffered from PPPD since it is a diagnosis of exclusion. An NPQ score can exceed the cutoff in other conditions, such as SCDS, which we presented here. The routine usage of NPQ in our clinic is to analyze inducing factors. Among the 3 factors, “movement” scores are the highest in our cases (Figure 3), whereas “visual stimulation” is usually characteristic of PPPD (12), which could be helpful in the differential diagnosis of these two conditions.

These positive outcomes persisted, with no signs of symptom aggravation or recurrence for a duration nearing 3.7 years (Figure 3). Historically, concerns have been raised regarding the durability of multilayer RWR, with failures documented as early as 6 months post-treatment (18, 19). This duration is considerably shorter than the longevity reported for other surgical approaches, such as the middle cranial fossa or transmastoid technique (20). The early failure of RWR is often ascribed to the potential for fascia graft degradation, which may undergo atrophy, resorption, or migration (21).

Addressing this issue, Wackym et al. enhanced the RWR procedure by incorporating fascia and cartilage, as well as denuding all mucosa around the RW niche using a CO_2_ laser to secure the materials. The procedure was described in much greater detail than in previous publications, as clarified in reference (6). We have further adapted this procedure. We refrained from drilling off the bony overhang of the niche; instead, we utilized the niche space to fill and stabilize with the first cartilage, positioning the perichondrium toward the RWM to minimize invasiveness to the cochlea and RW membrane. 0.25 mm cartilage fragments further stabilized the first cartilage. A thinned layer of connective tissue was applied to encase the first cartilage, extending over the exposed bone encircling the niche. Additional layers of thinly sliced cartilage were superimposed to avert migration. This stratified technique augmented the graft’s stability postoperatively, enabling extended-term outcomes. The follow-up period described in previous reports regarding the long-term outcomes of RWR was about 2 years, and more documentation of extended follow-up durations is needed (6).

This study carries several weaknesses. The present study is limited by the absence of a control group, either non-surgical or receiving sham surgery, which introduces the potential for a placebo effect on subjective symptoms. Regardless, our findings demonstrated that patients exhibited marked improvement post-surgery even among the 2 cases presenting chronic SCDS symptoms. This supports the inference that the observed gains are likely attributable to the surgical intervention.

In cases where RWR was performed for SCDS, it could elevate the threshold in the air-conduction testing as a surgical complication (7, 16, 22, 23). In Case 1, there was no exacerbation, while Case 2 exhibited a 20 dB exacerbation in the air-conduction testing postoperatively; however, the patient reported satisfaction due to the resolution of the debilitating vestibular dysfunction that was interfering with daily activities. Regarding cVEMP and oVEMP, postoperative testing revealed normalization of responses on the operated side after surgical plugging (24). Previous reports lacked detailed descriptions of postoperative VEMP changes in cases where RWR was performed. In our two cases, the enhanced responses of oVEMP did not normalize.

The RWR technique aims to alleviate symptoms associated with a third window by modifying the compliance of the round window (RW) rather than directly addressing the more challenging-to-access SC (6). The inner ear becomes less sensitive to sudden changes in sound and pressure without the dehiscence itself being repaired. It should be noted that this surgical procedure is aimed at alleviating vestibular symptoms and is not designed to improve the hearing component. Additionally, it may result in a decrease in conductive hearing. And that Medical Centers with low experience in the middle fossa approach technique should think over the RWR technique due to possible complications. Case 1 had an SPS-type dehiscence; the anatomical location of the SPS can make it challenging to maintain a visual field in the operative area and may increase the risk of surgical complications (25). Multilayer RWR is a preferable first choice, especially in SPS cases with a high risk of surgical complications with conventional methods.

## Conclusion

We successfully treated two cases of SCDS with the multilayer RWR technique, resulting in significant improvement in subjective symptoms and quality of life during the early postoperative period, with these benefits lasting for at least approximately 3.7 years. The primary limitation of this study is that it includes only two cases, which may limit the conclusiveness of our findings. Another limitation of this technique is the potential for no improvement or even deterioration of the conductive component of hearing, despite the alleviation of vestibular symptoms. However, RWR carries a lower risk of complications such as intracranial injury, cerebrospinal fluid leakage, sensorineural hearing loss, and infection than conventional methods. It can also effectively alleviate symptoms without requiring craniotomy. Considering these factors, the multilayer RWR technique can be a favorable choice for initial surgery in the treatment of SCDS.

## Data availability statement

The datasets presented in this study can be found in online repositories. The names of the repository/repositories and accession number(s) can be found in the article/supplementary material.

## Ethics statement

Written informed consent was obtained from the individual(s), and minor(s)’ legal guardian/next of kin, for the publication of any potentially identifiable images or data included in this article.

## Author contributions

MS: Writing – original draft, Writing – review & editing. HM: Investigation, Writing – review & editing. YT: Investigation, Writing – review & editing. KS: Investigation, Writing – review & editing. HK: Investigation, Writing – review & editing. MN: Writing – review & editing, Investigation. TI: Conceptualization, Supervision, Writing – review & editing.

## References

[1] Wackym PA Wood SJ Siker DA Carter DM. Otic capsule dehiscence syndrome: superior semicircular canal dehiscence syndrome with no radiographically visible dehiscence. Ear Nose Throat J. (2015) 94:E8–9. doi: 10.1177/014556131509400802, PMID: 26322461

[2] Bi WL Brewster R Poe D Vernick D Lee DJ Eduardo Corrales C. Superior semicircular canal dehiscence syndrome. J Neurosurg. (2017) 127:1268–76. doi: 10.3171/2016.9.JNS16503 28084916

[3] Minor LB Solomon D Zinreich JS Zee DS. Sound-and/or pressure-induced vertigo due to bone dehiscence of the superior semicircular canal. Arch Otolaryngol Neck Surg. (1998) 124:249. doi: 10.1001/archotol.124.3.249 9525507

[4] Palma Diaz M Cisneros Lesser J Vega AA. Superior semicircular canal dehiscence syndrome – diagnosis and surgical management. Int Arch Otorhinolaryngol. (2017) 21:195–8. doi: 10.1055/s-0037-1599785 28382131 PMC5375705

[5] Wackym PA Balaban CD Zhang P Siker DA Hundal JS. Third window syndrome: surgical Management of Cochlea-Facial Nerve Dehiscence. Front Neurol. (2019) 10:1281. doi: 10.3389/fneur.2019.01281, PMID: 31920911 PMC6923767

[6] Wackym PA Balaban CD Mackay HT Wood SJ Lundell CJ Carter DM. Longitudinal cognitive and neurobehavioral functional outcomes before and after repairing otic capsule dehiscence. Otol Neurotol. (2016) 37:70–82. doi: 10.1097/MAO.0000000000000928, PMID: 26649608 PMC4674143

[7] Silverstein H Kartush JM Parnes LS Poe DS Babu SC Levenson MJ. Round window reinforcement for superior semicircular canal dehiscence: a retrospective multi-center case series. Am J Otolaryngol. (2014) 35:286–93. doi: 10.1016/j.amjoto.2014.02.016, PMID: 24667055

[8] Carey JP Minor LB Nager GT. Dehiscence or thinning of bone overlying the superior semicircular canal in a temporal bone survey. Arch Otolaryngol Neck Surg. (2000) 126:137. doi: 10.1001/archotol.126.2.137 10680863

[9] Jacobson GP Newman CW. The development of the dizziness handicap inventory. Arch Otolaryngol Head Neck Surg. (1990) 116:424–7. doi: 10.1001/archotol.1990.01870040046011 2317323

[10] Yardley L Barker F Muller I Turner D Kirby S Mullee M. Clinical and cost effectiveness of booklet based vestibular rehabilitation for chronic dizziness in primary care: single blind, parallel group, pragmatic, randomised controlled trial. BMJ. (2012) 344:e 2237. doi: 10.1136/bmj.e2237, PMID: 22674920 PMC3368486

[11] Yardley L Masson E Verschuur C Haacke N Luxon L. Symptoms, anxiety and handicap in dizzy patients: development of the vertigo symptom scale. J Psychosom Res. (1992) 36:731–41. doi: 10.1016/0022-3999(92)90131-K, PMID: 1432863

[12] Yardley L Donovan-Hall M Smith HE Walsh BM Mullee M Bronstein AM. Effectiveness of primary care–based vestibular rehabilitation for chronic dizziness. Ann Intern Med. (2004) 141:598–605. doi: 10.7326/0003-4819-141-8-200410190-00007, PMID: 15492339

[13] Yagi C Morita Y Kitazawa M Nonomura Y Yamagishi T Ohshima S. A validated questionnaire to assess the severity of persistent postural-perceptual dizziness (PPPD): the Niigata PPPD questionnaire (NPQ). Otol Neurotol. (2019) 40:e747–52. doi: 10.1097/MAO.0000000000002325, PMID: 31219964 PMC6641087

[14] Ikezono T Matsumura T Matsuda H Shikaze S Saitoh S Shindo S. The diagnostic performance of a novel ELISA for human CTP (Cochlin-tomoprotein) to detect perilymph leakage. PLoS One. (2018) 13:e0191498. doi: 10.1371/journal.pone.0191498, PMID: 29377910 PMC5788340

[15] Pfammatter A Darrouzet V Gartner M Somers T Dinther JV Trabalzini F. A superior semicircular canal dehiscence syndrome multicenter study: is there an association between size and symptoms? Otol Neurotol. (2010) 31:447–54. doi: 10.1097/MAO.0b013e3181d27740 20118818

[16] Chemtob RA Noij KS Qureshi AA Klokker M Nakajima HH Lee DJ. Superior canal dehiscence surgery outcomes following failed round window surgery. Otol Neurotol. (2019) 40:535–42. doi: 10.1097/MAO.0000000000002185, PMID: 30870372

[17] Ward BK Van de Berg R Van Rompaey V Bisdorff AR HT E Welgampola MS Society ICVD Proposal Superior Semicircular Canal Dehiscence Syndrome (SCDS). (2016). [in press].

[18] Thomeer H Bonnard D Castetbon V Franco-Vidal V Darrouzet P Darrouzet V. Long-term results of middle fossa plugging of superior semicircular canal dehiscences: clinically and instrumentally demonstrated efficiency in a retrospective series of 16 ears. Eur Arch Otorrinolaringol. (2016) 273:1689–96. doi: 10.1007/s00405-015-3715-5, PMID: 26205152 PMC4899492

[19] Mau C Kamal N Badeti S Reddy R Ying YLM Jyung RW. Superior semicircular canal dehiscence: diagnosis and management. J Clin Neurosci. (2018) 48:58–65. doi: 10.1016/j.jocn.2017.11.019 29224712

[20] Alkhafaji MS Varma S Pross SE Sharon JD Nellis JC Santina CCD. Long-term patient-reported outcomes after surgery for superior canal dehiscence syndrome. Otol Neurotol. (2017) 38:1319–26. doi: 10.1097/MAO.0000000000001550, PMID: 28902804

[21] Shaia WT Diaz RC. Evolution in surgical management of superior canal dehiscence syndrome. Curr Opin Otolaryngol Head Neck Surg. (2013) 21:497–502. doi: 10.1097/MOO.0b013e328364b3ff, PMID: 23989599

[22] Silverstein H Van Ess MJ. Complete round window niche occlusion for superior semicircular canal dehiscence syndrome: a minimally invasive approach. Ear Nose Throat J. (2009) 88:1042–56. doi: 10.1177/014556130908800808, PMID: 19688714

[23] Succar EF Manickam PV Wing S Walter J Greene JS Azeredo WJ. Round window plugging in the treatment of superior semicircular canal dehiscence. Laryngoscope. (2018) 128:1445–52. doi: 10.1002/lary.26899, PMID: 28990655

[24] Rinaldi V Portmann D. Vestibular-evoked myogenic potentials after superior semicircular canal obliteration. Rev Laryngol Otol Rhinol. (2011) 132:85–7. 22416487

[25] McCall AA McKenna MJ Merchant SN Curtin HD Lee DJ. Superior canal dehiscence syndrome associated with the superior petrosal sinus in pediatric and adult patients. Otol Neurotol. (2011) 32:1312–9. doi: 10.1097/MAO.0b013e31822e5b0a, PMID: 21918420

